# Epidemiology of cleft lip and palate in Bhutan, 2015–2022

**DOI:** 10.1186/s12903-024-05177-7

**Published:** 2024-11-15

**Authors:** Karma Tobgyel, Prakriti Rai, Kuenga Choden, Tshewang Gyeltshen

**Affiliations:** 1 Department of Dentistry, Jigme Dorji Wangchuck National Referral Hospital, Taba, Bhutan; 2 Department of Advanced Prosthodontics, Tokyo Medical and Dental University, Tokyo, Japan; 3 Department of Global Health Policy, School of International Health, Graduate School of Medicine, University of Tokyo, Tokyo, Japan; 4 Division of Population Data Science, National Cancer Centre, Institute for Cancer Control, Tsukiji, Tokyo Japan

**Keywords:** Orofacial clefts, Cleft lip and palate, Bhutan, Birth defect surveillance

## Abstract

**Background:**

The epidemiology of cleft lip (CL) and cleft palate (CLP) has not previously been described in the context of the Bhutan and Bhutanese populations. Using National Birth Defects Surveillance Data and other vital statistics, we present the comprehensive epidemiology of the cleft lip and palate in Bhutan.

**Methodology:**

The National Birth Defects Surveillance Data Registry from 2015 to 2022 is reviewed retrospectively, covering 8 years of birth defect surveillance in the country from records maintained with three referral hospitals in the country. The baseline prevalence and incidence of cleft lip and palate have been presented over the years. The incidence of cleft lip and palate was defined as the number of cases per 1000 live births. We used Poisson’s regression to compute the incidence of cleft lip and palate. Pearson chi-square tests (χ2) were used to examine the associations of maternal and child characteristics with cleft lip and palate.

**Results:**

A total of 1401 newborns with various birth defects were born among the 89,078 live births from 2015 to 2022 in Bhutan. Of these, 122 (8.7%) constituted orofacial clefts. The prevalence of orofacial clefts tended to increase, with a period prevalence of 1.37 per 1000 live births. There were more cases in males (72) than in females (50). The incidence rate ratio ranged from 1.2 to 2.0 compared with the 2015 baseline year, indicating increased rates over time.

**Conclusion:**

Orofacial clefts constituted 8.7% of total birth defects and 1.37 per 1000 live births over the years. The increasing prevalence trends and incidence rate ratios over the years underscore the importance of ongoing surveillance and interventions to address the burden of orofacial clefts in Bhutan.

**Clinical trial number:**

Not applicable.

## Introduction

The cleft lip and palate (CLP) is the most common congenital craniofacial anomaly globally, with an estimated prevalence of 1 in 700 live births [1]. CLP presents either as an isolated cleft lip or cleft lip with palate involvement. The World Health Organization estimates that the global birth incidence of CLP is approximately 15.37 per 10,000 live births. The prevalence of CLP has been found to be highest among the Asian population at 0.82–4.04 per 1000 live births [2, 3]. With a relatively small population of 0.7 million people, the cleft and its prevalence have not been described before in the context of Bhutanese population.

CLP, a congenital deformity, is caused by abnormal facial development during intrauterine development. It can occur on the lip only (CL), the palate (CP) or can involve the lip and palate both (CLP) [4]. CLP can result in both aesthetic and functional challenges. Newborns with CLP often experience difficulties such as speech anomalies, swallowing issues, feeding challenges, and recurrent ear infections [5, 6]. The impact of CLP extends beyond physical and functional impairments. It strongly negatively affects individuals’ psychosocial behaviours, thus affecting their quality of life [6–8]. The impact of CLP on development and quality of life of affected individuals and their families is substantial, even in environments where specialized early care is readily available [9, 10].

The CLP develop in utero when the frontonasal process fails to fuse with the two lateral maxillary processes and the palatine processes at five to ten weeks of gestation [11, 12]. Genetic, environmental, and nutritional factors are reported to contribute to orofacial cleft development [13–15]. The intake of folic acid and micronutrients both during pregnancy and during the preconception period has been shown to play a preventative role in the development of cleft conditions [16].

Bhutan has achieved remarkable success in improving maternal and child health over the years [17]. Through integrated antenatal care services, the country has focused on preventing birth defects, including CLP defects. With a commitment to enhancing maternal and neonatal health, Bhutan continues to strengthen its efforts in this area. Despite these advancements, the prevalence and incidence of CLP within the Bhutanese population have not been previously reported. This study aims to provide a comprehensive analysis of the baseline prevalence and incidence of cleft lip and palate among the Bhutanese population from 2015 to 2022.

## Methodology

### Data source

We used data from the National Birth Defects Surveillance Data Registry maintained by the Jigme Dorji Wangchuck National Referral Hospital, Thimphu, Bhutan, from 2015 to 2022, which is maintained by the Pediatric Department of the hospital. These surveillance data are considered nationally representative, as all newborn defects in the country are reported. Vital statistics such as total live births in the country for each year were obtained from the National Statistics Bureau [18, 19]. Demographic characteristics such as birthweight, sex, maternal age, and paternal age have been described where the data were allowed. The WHO International Classification of Diseases (11th Revision, ICD-11) is used to identify the cleft type. The ICD code of Cleft Lip is Q36, that of Cleft Palate is Q35, and that of Cleft Lip and palate is Q37.

### Statistical analysis

We used proportions to present total cases by number of birth defects and total live births per year and calculated the prevalence per 1000 live births. We used Poisson’s regression to compute the incidence of CLP over the years. Pearson chi-square tests (χ2) were used to examine the associations of maternal and child characteristics with CLP. All the statistical analyses were performed via R version 4.4.0 [20].

## Results

A total of 122 newborns with clefts were born in Bhutan between 2015 and 2022 (Table 1). The majority of cleft newborns (84.4%) were born at term (37–42 weeks). The proportion of preterm births increased significantly over time, with the highest percentage (40.0%) occurring in 2022 (*p* < 0.001). No significant difference in sex distribution was observed across the years (*p* = 0.697), although 59.0% of the newborns with cleft were male, and 41.0% were female. The age distribution of mothers with newborn clefts varied significantly across the years (*p* = 0.004). The most common age group was 25–29 years (29.5%), followed by 20–24 years (24.6%) and 30–34 years (21.3%). The proportion of low birthweight (LBW) among the newborns have increased over time, with the highest percentage (46.7%) occurring in 2022. However, the differences in birthweight distribution across the years were not statistically significant (*p* = 0.308).

**Table 1:** Maternal, paternal and child characteristics; epidemiology of cleft lip and palate in Bhutan, 2015–2022

	2015 (*N* = 10)	2016 (*N* = 12)	2017 (*N* = 16)	2018 (*N* = 17)	2019 (*N* = 20)	2020 (*N* = 16)	2021 (*N* = 16)	2022 (*N* = 15)	Total (*N* = 122)	*p* value*
**Gestational Age**										< 0.001
Preterm	-	1 (8.3%)	-	2 (11.8%)	4 (20.0%)	1 (6.2%)	1 (6.2%)	6 (40.0%)	15 (12.3%)	
Term (37–42 Weeks)	10 (100.0%)	11 (91.7%)	16 (100.0%)	11 (64.7%)	16 (80.0%)	15 (93.8%)	15 (93.8%)	9 (60.0%)	103 (84.4%)	
Above 42 Weeks	-	-	-	4 (23.5%)	-	-	-	-	4 (3.3%)	
**Sex**										0.697
Female	3 (30.0%)	4 (33.3%)	8 (50.0%)	7 (41.2%)	6 (30.0%)	7 (43.8%)	6 (37.5%)	9 (60.0%)	50 (41.0%)	
Male	7 (70.0%)	8 (66.7%)	8 (50.0%)	10 (58.8%)	14 (70.0%)	9 (56.2%)	10 (62.5%)	6 (40.0%)	72 (59.0%)	
**Mother’s Age**										0.004
Below 20 Years	2 (20.0%)	-	2 (12.5%)	-	-	-	-	-	4 (3.3%)	
20–24 Years	3 (30.0%)	5 (41.7%)	9 (56.2%)	3 (17.6%)	3 (15.0%)	3 (18.8%)	2 (12.5%)	2 (13.3%)	30 (24.6%)	
25–29 Years	-	6 (50.0%)	5 (31.2%)	7 (41.2%)	6 (30.0%)	6 (37.5%)	3 (18.8%)	3 (20.0%)	36 (29.5%)	
30–34 Years	2 (20.0%)	-	-	4 (23.5%)	5 (25.0%)	2 (12.5%)	7 (43.8%)	6 (40.0%)	26 (21.3%)	
35–39 Years	2 (20.0%)	1 (8.3%)	-	-	5 (25.0%)	4 (25.0%)	2 (12.5%)	2 (13.3%)	16 (13.1%)	
>= 40 Years	1 (10.0%)	-	-	3 (17.6%)	1 (5.0%)	1 (6.2%)	2 (12.5%)	2 (13.3%)	10 (8.2%)	
**Father’s Age**										< 0.001
20–24 Years	3 (30.0%)	6 (50.0%)	2 (12.5%)	3 (17.6%)	1 (5.0%)	-	-	-	15 (12.3%)	
25–29 Years	2 (20.0%)	3 (25.0%)	7 (43.8%)	6 (35.3%)	-	-	-	-	18 (14.8%)	
30–34 Years	1 (10.0%)	2 (16.7%)	4 (25.0%)	3 (17.6%)	-	-	-	-	10 (8.2%)	
35–39 Years	2 (20.0%)	1 (8.3%)	3 (18.8%)	2 (11.8%)	1 (5.0%)	-	-	-	9 (7.4%)	
Above 40 Years	2 (20.0%)	-	-	3 (17.6%)	-	-	-	-	5 (4.1%)	
No Info	-	-	-	-	18 (90.0%)	16 (100.0%)	16 (100.0%)	15 (100.0%)	65 (53.3%)	
**Birthweight**										0.308
>= 2500 g	8 (80.0%)	11 (91.7%)	14 (87.5%)	12 (70.6%)	14 (70.0%)	13 (81.2%)	13 (81.2%)	8 (53.3%)	93 (76.2%)	
LBW	2 (20.0%)	1 (8.3%)	2 (12.5%)	5 (29.4%)	6 (30.0%)	3 (18.8%)	3 (18.8%)	7 (46.7%)	29 (23.8%)	

*Chi-square test

During the study period, a total of 72 male and 50 female newborns were diagnosed with cleft lip and palate, resulting in an overall incidence of 1.37 cases per 1,000 live births. The highest prevalence was observed in 2019, with a rate of 1.96 cases per 1,000 live births. The incidence of cleft lip and palate in newborns has shown an increasing trend in recent years, increasing from 1.20 cases per 1,000 live births in 2016 to 2.00 cases per 1,000 live births in 2019 and subsequently declining to 1.50 cases per 1,000 live births in 2022. The total number of live births each year and their prevalence and incidence rate ratios are presented in Table 2.

**Table 2:** Prevalence and Incidence of Clefts in Bhutan; Epidemiology of Cleft lip and Palate in Bhutan 2015–2022

Year	Cleft Lip and Palate				Number of Birth Defects*	Total Number of Live Births		
	Male	Female	Prevalence*	IRR (95% CI) **		Male	Female	Total
2015	7	3	10 (0.75)	-	117 (8.83)	-	-	13248
2016	8	4	12 (1.15)	1.20 (0.52–2.84)	153 (14.63)	5400	5057	10457
2017	8	8	16 (1.51)	1.60 (0.74–3.65)	287 (27.04)	5423	5189	10612
2018	10	7	17 (1.50)	1.70 (0.79–3.85)	211 (18.56)	5753	5615	11368
2019	14	6	20 (1.96)	2.00 (0.96–4.46)	230 (22.58)	5191	4995	10186
2020	9	7	16 (1.56)	1.60 (0.74–3.65)	120 (11.27)	5266	4974	10240
2021	10	6	16 (1.49)	1.60 (0.74–3.65)	175 (16.27)	5452	5303	10755
2022	6	9	15 (1.23)	1.50 (0.68–3.45)	108 (8.84)	-	-	12212
TOTAL	72	50	122 (1.37)		1401 (15.73)	32485*	31133*	89078

*Prevalence per 1000 live births

**Incidence Rate Ratio per 1000 live births

**Fig. 1:**
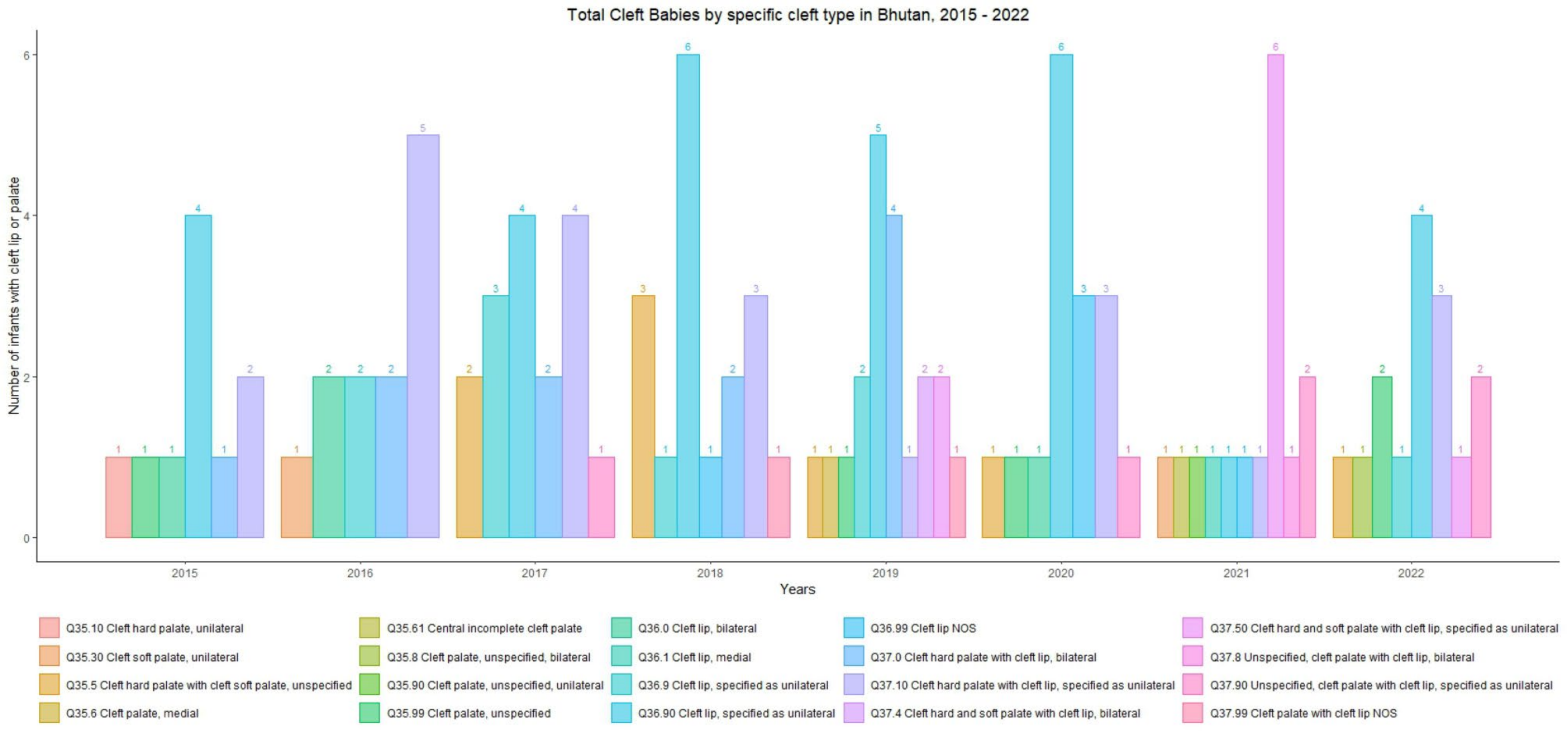
Prevalence of Cleft Lip and Palate by types, 2015–2022, Bhutan

**Fig. 2:**
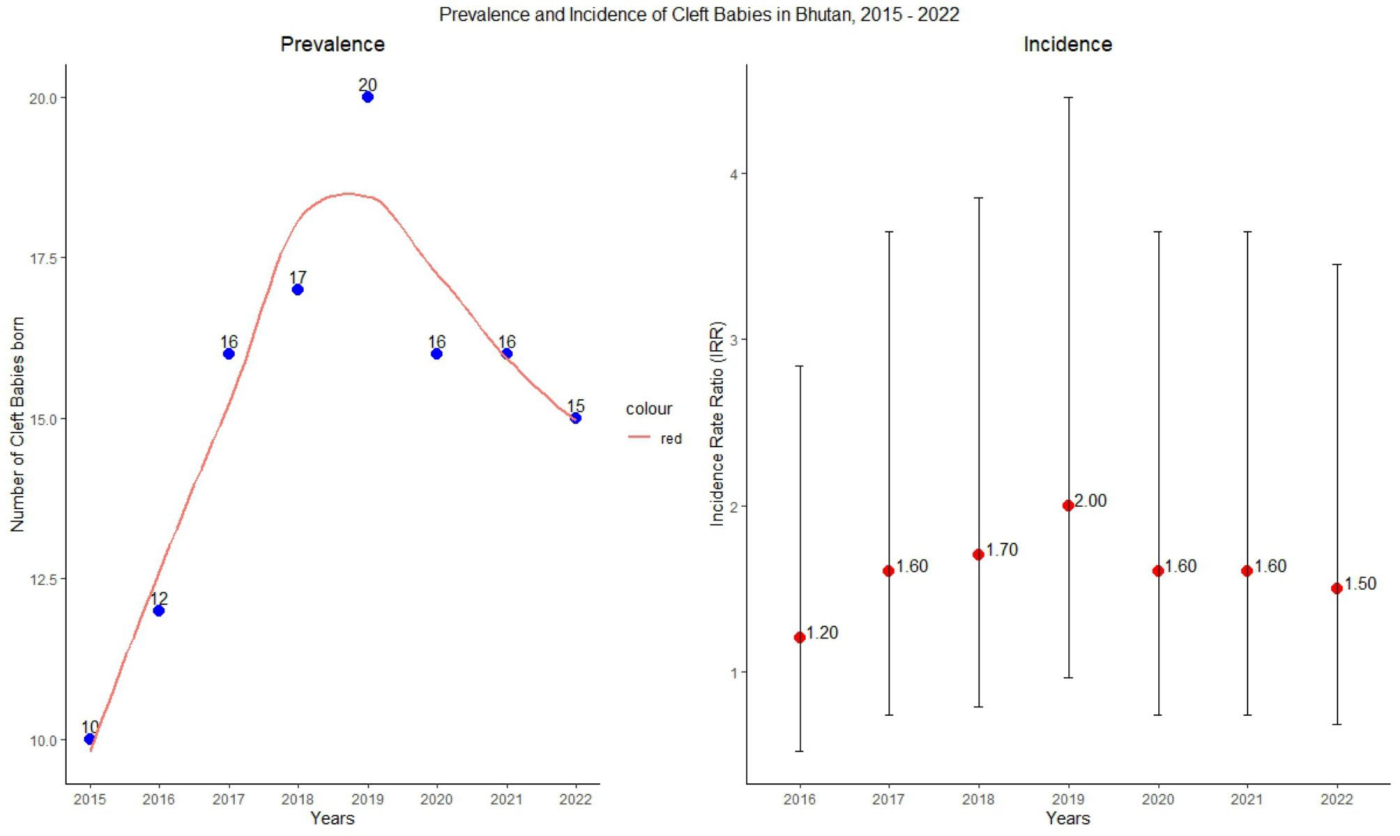
Prevalence and Incidence of Cleft Lip and Palate, 2015–2022, Bhutan

Most of the clefts observed over the study period were unilateral clefts. The most common category was unilateral cleft lip (ICD Q36.90), constituting 30 occurrences (24.59%). The second most common category was unilateral cleft hard palate with cleft lip (ICD Q37.10), with 22 occurrences (18.03%). Unilateral cleft hard and soft palate with cleft lips (ICD Q37.50) accounted for 9 occurrences (7.38%). The remaining cases are distributed among various other forms of cleft, both specified and unspecified, including bilateral and medial clefts. The distribution of newborn clefts by type and year is shown in Figs. 1, 2.

## Discussion

Orofacial clefts cause significant health burdens in addition to affecting an individual’s quality of life [21]. Between 2015 and 2022, a total of 122 newborns with cleft lip and palate were born in Bhutan, with an overall incidence of 1.37 cases per 1,000 live births. Most of these newborns (84.4%) were born at term. The incidence of newborns with cleft has shown an increasing trend, peaking in 2019 at 1.96 cases per 1,000 live births before declining to 1.50 cases per 1,000 live births in 2022. Males were generally more affected (59.0%).

The global incidence of cleft lip and palate is reported to be 1.08% [22]. Our study consistently revealed that the incidence of orofacial clefts in Bhutan is lower each year than the global incidence. Compared with our findings, the prevalence of orofacial clefts in neighboring South Asian countries varies: 1.64 per 1000 live births per year for Nepal [23], 1.3 per 1000 live births in India [24], and 0.83 per 1000 live births in Sri Lanka [25]. This study revealed that the orofacial cleft prevalence in Bhutan was similar to that reported by Cooper et al. for Japanese (1.34), Chinese (1.30) and other Asian races (1.44) [26]. A study on white race among the Czech population by Urbanova et al., reported 1.64 per 1000 live births [27].

The type and frequency of orofacial clefts may vary by population and racial background as discussed. While Bhutan’s incidence rates align closely with those of other Asian populations the distribution of cleft types varies. The differences in genetic predispositions and environmental factors can contribute to these variations and distribution.

As the first study to report the epidemiology of orofacial clefts in Bhutan, we identify several factors contributing to the increasing trends in cleft cases. One significant factor is the country’s commitment to providing free access to diagnostic services for pregnant women, supported by various policies and effective implementation strategies. For example, the National Child Health Strategy and the Bhutan Every Newborn Action Plan, introduced in 2016, have played crucial roles in enhancing prenatal care and early detection [28]. Second, the robust implementation of a birth defect surveillance system across the country, with support from the World Health Organization, has significantly enhanced the detection of these conditions.

An earlier study reported that male fetuses are more sensitive to environmental stress, which may lead to birth defects [29]. Similarly, we also found that male sex was more strongly associated with orofacial clefts than female sex was. This finding is consistent with an earlier report showing male dominance in a genetic study of 207 cases of oral clefts [30]. Similar reports of male dominance have also been reported in Finland, America, and France [27, 30–32].

### Limitations

Our study did not examine the determinants of orofacial clefts because of data limitations. The birth defect surveillance data were collected specifically for monitoring birth defects, and the homogeneity of the data made deterministic analysis unfeasible. To explore the broader epidemiological determinants of orofacial clefts, further studies are necessary.

Bhutan boasts a robust Mother and Child Health (MCH) tracking system, supported by surveillance sentinels distributed across all health centres in the country. Nonetheless, our study may be limited by potential underreporting, which is a common challenge associated with surveillance data. Despite this limitation, Bhutan’s relatively small population and the nation’s excellent access to healthcare services enhance the generalizability of our findings to the entire country. It is important, however, to exercise caution when interpreting our results to account for any inherent data limitations.

## Conclusion

Our findings highlight the increasing trend of orofacial clefts in Bhutan, which demands urgent attention and programmatic action. Given the increasing prevalence, there is a pressing need to establish a uniform birth registry, such as the SEARO-Newborn and Birth Defects Database, across all hospitals in the country.

## Data Availability

The primary data which is part of the WHO SEARO Surveillance for Birth Defects is not available for public use. However, this can be made available from the corresponding author upon reasonable request. The aggregated data, and the codes used to generate the results and analysis are provided in the personal archive of the corresponding author made available at: https://github.com/tgyeltshen/Epidemiology-of-cleft-lip-and-palate-in-Bhutan.

## Abbreviations

CLP: Cleft Lip and Palate
CP: Cleft Palate
CL: Cleft Lip
ICD: International Classification of Diseases
REBH: Research Ethics Board of Health
WHO: World Health Organization
SEARO: Office of the WHO Southeast Asia Region
